# Adverse events reporting by obstetric units in Norway as part of their quality assurance and patient safety work: an analysis of practice

**DOI:** 10.1186/s12913-021-06956-6

**Published:** 2021-09-08

**Authors:** Lars T. Johansen, Geir Sverre Braut, Ganesh Acharya, Jan Fredrik Andresen, Pål Øian

**Affiliations:** 1 Department for Specialized Health Services, Norwegian Board of Health Supervision, PO Box 231 Skøyen, 0213 Oslo, Norway; 2 Stavanger University Hospital, Stavanger, Norway; 3 Western Norway University of Applied Sciences, Sogndal, Norway; 4 Women’s Health and Perinatology Research Group, Department of Clinical Medicine, UiT-The Arctic University of Norway, Tromsø, Norway; 5 Department of Obstetrics and Gynecology, University Hospital of North Norway, Tromsø, Norway; 6 Division of Obstetrics and Gynecology, Department of Clinical Science, Intervention and Technology, Karolinska Institute and Center for Fetal Medicine, Department of Women’s Health, Karolinska University Hospital, Stockholm, Sweden

**Keywords:** Supervision, Adverse event, Asphyxia, Shoulder dystocia, Postpartum hemorrhage, Reporting culture, Obstetric care unit, Failure of treatment.

## Abstract

**Background:**

The Norwegian Board of Health Supervision aims to contribute to the improvement of quality and patient safety in the healthcare services. Planned audits were performed to investigate how 12 selected Norwegian obstetric units reported and analyzed adverse events as the part of their quality assurance and patient safety work.

**Methods:**

Serious adverse events coded as birth asphyxia, shoulder dystocia and severe postpartum hemorrhage that occurred during 2014 (the most recent year for which the quality assured data were available) were obtained from the Medical Birth Registry of Norway. The obstetric units were asked to submit medical records, internal adverse events reports, and their internal guidelines outlining which events should be reported to the quality assurance system. We identified the adverse events at each obstetric unit that were reported internally and/or to the central authorities. Two obstetricians carried out an evaluation of each event reported.

**Results:**

Five hundred fifty-three serious adverse events were registered among 17,323 births that took place at the selected units. Twenty-one events were excluded because of incorrect coding or missing information. Eight events were registered in more than one category, and these were distributed to the category directly related to injury or adverse outcome. Nine of twelve (75 %) obstetric units had written guidelines describing which events should be reported. The obstetric units reported 49 of 524 (9.3 %) serious adverse events in their internal quality assurance system and 39 (7.4 %) to central authorities. Of the very serious adverse events, 29 of 149 (19.4 %) were reported. Twenty-three of 49 (47 %) reports did not contain relevant assessments or proposals for improving quality and patient safety.

**Conclusions:**

This study showed that adverse event reporting and analyses by Norwegian obstetric units, as a part of quality assurance and patient safety work, are suboptimal. The reporting culture and compliance with guidelines need to be improved substantially for better safety in patient care, risk mitigation and clinical quality assurance.

## Background

Good quality healthcare services should provide health benefits, be safe and take care of users’ wishes and needs [1]. This also applies in maternity care where the mother or child may get injured as a result of malpractice and substandard care [2]. There are several factors that can contribute to improving quality in obstetric care. Healthcare professionals must possess appropriate knowledge, skills and attitude, as well as keep their competencies up-to-date through continuous learning and practical skills training. In this way, they will be able to efficiently manage emergencies and appropriately handle adverse events. They must also be able to work in teams and communicate clearly. Leaders in obstetric units have a responsibility to ensure that professional and organizational guidelines are developed, revised and made known to the health personnel engaged in patient care. Finally, it is important to establish a culture of transparently reporting and learning from serious adverse events [3–6].

In the recent years, healthcare systems in many countries, including Norway, have increased their focus on quality assurance and patient safety. Some have implemented patient safety programs to reduce the number of adverse events [7, 8]. Clinical leaders have a special responsibility to build a culture of patient safety that can help strengthen and improve services [9].

Several countries have established sanction-free reporting systems in line with recommendations from the WHO (www.who.int) and the Council of Europe (www.coe.int). Some have national reporting systems, whereas others rely on local or regional ones. Some countries have voluntary notification systems (USA, New Zealand, Denmark) and in others this may be required by law (Norway, Sweden). There may also be different criteria as to what is mandatory in terms of reporting. Denmark (www.stps.dk) and England (www.england.nhs.uk) have established systematic reporting at a national level.

There are few international studies dealing with the reporting of serious events in obstetric care. A study from New Zealand showed a significant underreporting of adverse events, where a mother or child died or was injured, to quality registers [10]. In the UK, a national reporting system for adverse events was established in 2003 (NRLS), but the data related to obstetric care show that the events are not always correctly reported [11].

In Norway, it is mandatory to report all births to the Medical Birth Registry of Norway (MBRN). In addition, legislation requires the reporting of serious events to the central authorities. A review of supervisory cases that included serious adverse events in obstetric care showed that only 39.1 % of the events were reported by the obstetric units [2].

Adverse events should be reviewed and analyzed by every single unit, to see if routines and practices should be changed. This is regarded as an important part of quality assurance and patient safety work in Norway (the Specialist Health Services Act § 3-4a and the Health Care Supervision Act § 3). In 2012, the reporting system to central authorities was changed so that the notifications were to be sent to a sanction-free authority, the Norwegian Directorate of Health (Specialist Health Services Act § 3–3). At the same time, a scheme was introduced where the most serious events (deaths and very serious injuries) were to be notified to The Norwegian Board of Health Supervision (NBHS), a governmental supervisory authority for child welfare, health and social services in Norway (Specialist Health Services Act § 3-3a).

Causal analyses following adverse events should try to provide answers to: what happened, why it happened, and what can be done to prevent similar events from happening again. Retrospective analyses within other sectors, e.g. aviation, have been used for decades, but are now also implemented after adverse events in the health service. In the USA, UK and Denmark, hospitals must carry out such an analysis in the wake of adverse events [12, 13]. Risk analyses have attracted increasing research interest, also in obstetrics [13–15].

Several studies, in which the quality of obstetric practice has been assessed, have been based on serious events that were reported to supervisory authorities, patient injury systems, or processed in the judicial system [11, 16, 17]. These represent selected material that, to a small extent, describe the scope of adverse events and how often they are reported internally or to central authorities.

We hypothesized that although they comprise an important part of quality assurance and patient safety work, only a small proportion of adverse events are reported or analyzed by obstetric units. The aim of this study was to investigate whether adverse events in obstetric care were reported to central authorities or just to the obstetric unit’s own internal quality assurance and adverse event reporting system. We also wanted to see if the events reported internally were analyzed and assessed adequately by the obstetric units’ own quality and safety councils and if there were differences among small, medium and large obstetric units. In addition, we wanted to analyze if there was a difference in the number of events reported by the obstetric units that the supervisory authorities considered as high-risk and selected for closer surveillance, compared to the other units.

## Methods

This study is based on data collected by NBHS, by the authority of its supervisory mandate, during inspections of 12 selected obstetric units in Norway looking at three categories (birth asphyxia, shoulder dystocia and severe postpartum hemorrhage) of serious adverse obstetric events. These three categories are known to be associated with a substantial risk of serious adverse outcome for the mother or child. The inspection process, data collection and validation have been reported previously [18]. The study period was limited to include reported cases occurring between January 1 and December 31 2014, as the data from this year were the latest that were quality assured by the MBRN. The total number of obstetric units in Norway at this time was 49 (31 with < 1000 births per year, 11 with 1000–1999 births per year, and seven with ≥ 2000 births per year). Six of the units were chosen because the supervisory authorities had suspected that the obstetric care delivered at these units was potentially high-risk, and that there was an indication to survey their routines and practices. This risk assessment was based on the experiences gathered from previous inspections. Another six units were chosen randomly for comparison. In total, we evaluated six obstetric units with < 1000 births per year (small), four units with 1000–1999 births per year (medium) and two units with ≥ 2000 births per year (large).

The three categories of serious adverse events were chosen according to the following criteria as described previously [18]:

1. Birth asphyxia: singleton birth during which the baby died *in utero* after the mother’s admission to the obstetric unit, during birth, during the first 6 days after birth, or the infant had an Apgar score of < 7 after 5 min of birth and needed to be transferred to the neonatal intensive care unit.
2. Shoulder dystocia: vaginal singleton birth with difficult delivery of the shoulder and the newborn requiring admission to the neonatal intensive care unit or the infant with confirmed brachial plexus injury or fracture of humerus and/or clavicle.
3. Severe postpartum hemorrhage (PPH): vaginal singleton birth during which the mother bled more than 1500 millilitre (mL) within 24 h of birth and/or received blood transfusion.

Events with neonatal Apgar score < 5 after 5 min of birth, brachial plexus injuries or fractures, and hemorrhage ≥ 2500 mL, were defined as very serious adverse events.

On July 6, 2016, the NBHS requested information from the MBRN regarding the number of adverse events in these three categories of adverse events (birth asphyxia, shoulder dystocia, severe PPH) reported by each of the 12 obstetric units during the study period, based on the criteria described above.

Multiple pregnancies, births occurring before 36 weeks of gestation, and neonates with malformations (structural or chromosomal) were excluded.

On November 30, 2016, the NBHS requested copies of medical records of the cases from all 12 obstetrics units, along with the journal entries by midwives and obstetricians regarding the course of labor and delivery, as well as the reports from their own evaluation of the adverse events. In addition, we requested the institutions’ local guidelines regarding when and how to report adverse events.

All 12 obstetric units responded to the inquiry. Eleven units responded by January 6, 2017. The last unit replied on June 2, 2017, after a reminder.

The results were registered in a database designed for this study. For each adverse event, the obstetric unit, type of the event and method of delivery were recorded. We registered events reported to central authorities as well as those evaluated by the obstetric units locally. Two of the authors (L.T.J., P.Ø.), both senior consultant obstetricians, evaluated these reports. We evaluated whether the obstetric care was in accordance with standard clinical practice, based on Norwegian obstetric guidelines and international literature [19]. This evaluation was carried out independently by the two obstetricians. In case of discrepancy, a final decision was made after discussion. After completion of the audit, all the obstetric units that were evaluated received a detailed report on their own performance and recommendations from the NBHS regarding areas requiring improvements.

Data analysis was performed using IBM SPSS Statistics version 26.0. Quantitative data were grouped according to the size of the obstetric units and comparisons were made between the groups using chi-squared test. A *p*-value of < 0.05 was considered significant.

## Results

The prevalence of adverse events and the rate of occurrence of substandard care within each category of events in this study population have been reported previously [18]. The MBRN registered 553 serious adverse events within the three categories studied, among 17,323 deliveries in the 12 obstetric units. Twenty-one cases were excluded from analysis (incorrectly coded diagnosis in 10 cases and incomplete medical records in 11 cases). The cases (*n* = 8) that had the adverse events registered in more than one category were assigned to the category directly related to the injury or adverse outcome. Thus, a total of 524 cases with adverse events were included in the final analysis. The prevalence of adverse events within the categories evaluated was 3 %, of which 19.7 % (*n* = 103) had birth asphyxia, 11.4 % (*n* = 60) had shoulder dystocia or brachial plexus injury, and 68.9 % (*n* = 361) had severe PPH.

Nine (5.5 %) of 163 neonates died during or shortly after birth, 161 (98.7 %) neonates had to be treated in neonatal intensive care, and 33 (20.2 %) had a brachial plexus injury or fracture of humerus and/or clavicle. Of the 361 women who had severe PPH, 254 (70.3 %) had blood transfusion and 145 (40.1 %) had a prolonged hospital stay (> 3 days after vaginal birth).

According to the medical records 221 (42.1 %) patients received information about the birth process from a doctor or midwife, 112 (21.3 %) received a call or were examined again after discharge. It was further documented that seven (1.3 %) patients were informed about health care failure and five (0.9 %) about the possibility of applying for financial compensation from The Norwegian System of Compensation to Patients (NPE).

The obstetric units reported internally 27 of 112 (26 %) events with birth asphyxia, five of 60 (8.3 %) events with shoulder dystocia and brachial plexus injury, 17 of 361 (4.7 %) events with severe PPH. A total of 49 of 524 (9.3 %) events with serious adverse events, and 29 of 149 (19.4 %) of those with a very serious adverse events were reported in the internal quality assurance and adverse event reporting system. Table 1 shows the total number of adverse events and how many of them were reported internally in small, medium-sized and large obstetric care units. There were statistically significant differences between the three groups of obstetric care units.

**Table 1:** Number of adverse events (birth asphyxia, shoulder dystocia, severe postpartum hemorrhage) obtained from the Medical Birth Registry of Norway and events reported in the internal quality assurance and adverse event reporting system of obstetric units according to their size (small < 1000 births per year, medium 1000–1999 births per year, large ≥ 2000 births per year) in 12 Norwegian obstetric units in 2014

Size of maternity unit	Number of births at term	Number of adverse events	Number of adverse events per 1000 births	Number of adverse events reported	Number of adverse events reported per 1000 births	Number of adverse events related to events reported per 1000 births (%)
< 1000 births per year	2927	72	24.59 (72/2927)	7	2.39 (7/2927)	9.71 (2.39/24.59)
1000–1999 births per year	5907	188	31.82 (188/5907)	28	4.74 (28/5907)	14.89 (4.74/31.82)
≥2000 births per year	8489	264	31.09 (264/8489)	14	1.64 (14/8489)	5.27 (1,64/31.09)

Chi-square test: χ^2^ = 11,9, df = 2, *p* = 0.002

Table 2 shows the number of adverse events with very serious adverse events and how many of them were reported in the internal quality assurance and adverse event reporting system. There were statistically significant differences between the obstetric units grouped by size.

**Table 2:** Number of very serious adverse events (death or asphyxia with Apgar < 5 after 5 min, brachial plexus injury or fracture of clavicle/humerus, postpartum hemorrhage ≥2500 mL) obtained from the Medical Birth Registry of Norway and reported in the internal quality assurance and adverse event reporting system of obstetric units according to their size (small < 1000 births per year, medium 1000–1999 births per year, large ≥ 2000 births per year) in 12 Norwegian obstetric units in 2014

Size of maternity unit	Number of births per year at term	Number of adverse events	Number of adverse events per 1000 births	Number of adverse events reported	Number of adverse events reported per 1000 births	Number of adverse events related to events reported per 1000 births (%)
< 1000 births per year	2927	14	4.78 (14/2927)	2	0.68 (2/2927)	14.22 (0.68/4.78)
1000–1999 births per year	5907	52	8.80 (52/5907)	18	3.04 (18/5907)	34.54 (3.04/8.80)
>2000 births per year	8489	83	9.77 (83/8489)	9	1.06 (9/8489)	10.84 (1.06/9.77)

Chi-square test: χ^2^ = 11,8, df = 2, *p* = 0.003

The number of adverse events that were reported to central authorities pursuant to the Specialist Health Services Act § 3–3 was a total of 39 (7.4 %), including a total of eight (1.5 %) according to the Specialist Health Services Act § 3-3a.

Table 3 shows the number of adverse events reported by the 12 obstetric units in their internal quality assurance system divided into two groups; six units selected by the supervisory authorities as high-risk for closer surveillance and six randomly selected units (control group). There were no statistically significant differences in the number (%) of reported events in the two groups.

**Table 3:** Number of obstetric adverse events (birth asphyxia, shoulder dystocia, severe postpartum hemorrhage) obtained from the Medical Birth Registry of Norway and events reported and analyzed in the internal quality assurance and adverse event reporting system in 12 Norwegian obstetric units in 2014, grouped into those selected by the supervisory authorities for a closer surveillance (*n* = 6 units) and those randomly allocated to a control group (*n* = 6 units). Chi-squared test with Yates correction

Type of maternity unit	Total number of births at term	Total number of adverse events, n (%)	Adverse events reported, n (%)	Number of adverse events with adequate assessment, n (%)
Six maternity units selected by the supervisory authorities as high-risk	7720	215 (2.78)	15 (6.97)	6 (40.00 %)
Six maternity units allocated randomly as a control group	9603	309 (3.21)	32 (10.35)	17 (53.12 %)
Chi^2^, df		2.7324, 1	2.5609, 1	2.4781, 1
*p*-value		> 0.10	> 0.10	> 0.10

We assessed all 49 adverse events reported to the internal quality assurance and adverse event reporting system that were analyzed locally. In our assessment, the analyses were relevant in 26 (53.0 %) events. In the remaining 23 (47.0 %), there was insufficient information about the events in the report, the assessment of the birth process was inadequate, and/or what measures should be implemented to improve the quality of care and patient safety was not described.

Nine of 12 obstetric care units (75.0 %) had written guidelines describing which events were to be reported, but only four of 12 (33.3 %) had described how the reports were to be processed and followed up.

## Discussion

In this study, we found that the obstetric units in Norway reported less than 10 % of adverse events with birth asphyxia, shoulder dystocia or severe postpartum hemorrhage in their own internal quality assurance and adverse event reporting system, and only 7.4 % were reported further to the central authorities. Less than one fifth of the very serious events were reported. It is worrying that even in a highly developed, high-income country with a national universal healthcare insurance coverage, a patient injury compensation system and sanction-free mandatory reporting required by law, so few events are reported and analyzed as part of quality assurance and patient safety work. As transparent reporting and appropriate analyses of adverse events is a prerequisite for learning, risk management and preventing patient injuries, improving reporting culture is essential. In addition to laws and government directives, better education and training of healthcare professionals in patient safety and risk management, as well as improving awareness and providing incentives for transparent reporting, may be required to motivate and engage them in clinical quality assurance.

In this study, we wanted to take a closer look at serious adverse clinical events. An adverse event is usually defined as an accidental injury to the patient that occurs in connection with medical/surgical treatment. The injury can have different levels of severity such as death, permanent disability or extended hospital stay [20]. The supervisory authorities wanted to assess events that fell into these categories. They obtained information from MBRN on the number of patients who had birth asphyxia, shoulder dystocia or severe postpartum hemorrhage and verified events reported by evaluating the medical records of the patients in question from each obstetric care unit. To our knowledge, no studies with a similar design have previously been reported. The method had advantages since we were able to obtain information on all the serious clinical adverse events in each category.

Our study showed that only a very small fraction of severe adverse obstetric events was reported internally and to the central authorities, although the majority of these events should have been reported, either because there was a patient injury or because the events could have led to serious injury (cf. the Specialist Health Services Act, Chap. 3). The purpose of the notification scheme is for the healthcare institutions to learn from adverse events. Therefore, especially in the case of events where there was a failure, these should have been reported, reviewed and analyzed. We have previously shown that there was a failure in diagnoses and treatment in 56.2 % of the events in this material, but no significant differences were observed between small, medium and large birth units [18]. In that study, we also found that a greater number of adverse events occurred in the control group, rather than in the maternity units considered to be at high-risk by the supervisory authorities. The supervisory authorities´ assessments of which units to consider as high-risk should be based on several parameters, not only on their experiences from previous inspections.

According to Norwegian law, these events should have been reported, but the text of the law is open to a certain discretionary assessment of whether the events must be reported or not. It is important to note that most obstetric care units (75 %) had their own guidelines for what events that should be reported. However, we have previously shown that the guidelines are not followed to a great extent [18, 21]. Furthermore, even among the very serious adverse events, which should have been reported in accordance with Norwegian law (Specialist Health Services Act §§ 3–3 and 3-3a) and national guidelines [19], less than one fifth were reported. Thus, there was significant under-reporting, and our findings are in concordance with other studies [22–24].

The survey also shows that there are statistically significant differences in reporting frequency between different sized obstetric units. It is striking that the reporting frequency seems to be highest in the medium-sized units. However, our study design does not make it possible to deduce any information about the reasons for these differences. Nevertheless, it might be reasonable to assume that the differences are related to the institutions’ organizational culture and clinical governance.

The secondary aim of our current study also was to evaluate if there were differences in reporting of adverse events between units selected by the supervisory authorities for closer surveillance and those randomly allocated as a control group. There were no significant differences between the two groups. This may indicate that the size of the obstetric units had the greatest significance for whether serious adverse events were reported, not the units considered as high-risk.

In Norway, anonymized reporting was introduced in 2012, which led to an increase in the number of events reported to central authorities [25]. However, our findings show that there are still many adverse obstetric events that are not reported. The data, which central authorities receive through the reporting schemes, therefore provide little information about the extent of adverse events in obstetric care and the types of events that occur [10, 11]. Several measures are needed to ensure that adverse events are reported correctly, transparently and comprehensively. Healthcare personnel must not only be familiar with the legal text that describes what needs to be reported, but must also know and follow the national guidelines from the professional society of obstetrics and gynecology [19].

Several studies have shown an underreporting of adverse events [22], including in obstetric units [2, 10, 23]. There may be several reasons for underreporting. The units that do not have a traditional culture of reporting and learning from serious events are less likely to have midwives and obstetricians reporting serious events [22]. Some obstetricians fail to report because they feel guilt or shame, others because they fear punishment [24]. It has been shown that reporting culture may be different among different healthcare professionals. Midwives seem to report adverse events more often than doctors, although often these are events without serious injury [26].

In our study, less than half of the reports contained relevant assessments and analyses of the adverse events or suggestions for improvements. In Norway, the incidents are assessed by the hospital’s quality assurance council. Members of this council consists of different healthcare professionals who are not always familiar with obstetric assessments. In our opinion, it was possible to make adequate assessment of the incidents if all information available in the medical record had been assessed by health personnel with the right competence.

According to Norwegian law, patients or relatives must receive information (Patient and User Rights Act § 3 − 2) about adverse events: what happened and what measures can be taken to prevent similar events from happening again. In addition, they must be informed about the possibility of applying for financial compensation. Our review of medical records showed that less than half received information about the birth process and only 0.9 % were informed about the possibility of applying for financial compensation to the Norwegian System of Compensation to Patients (NPE), a government agency under the Ministry of Health and Care Services (https://www.npe.no/en/). However, as our findings were based on the information obtained from medical records, it is not possible to rule out that this information was provided without being recorded. Sound information and good communication, which also mentions failures, help to create and build trust in health professionals. At the same time, it can help to facilitate the patients’ own processing of a traumatic birth process [27].

### Strengths and limitations

The strength of our study is that the information from MBRN made it possible to get a comprehensive overview of all adverse events in the three categories studied in each obstetric unit. The implementation of a planned inspection by to the Norwegian Board of Health Supervision ensured that all patient records and non-conformance reports were sent to the supervising authority. The weakness of the study is that the material is relatively small, especially the number of very serious adverse events. This means that the statistical analyses must be interpreted with caution. The data are from 2014, and may not reflect the current situation in Norway as there might have been changes in routines and practices regarding which incidents are to be reported and analyzed.

## Conclusions

This study shows that adverse event reporting by Norwegian obstetric units, as a part of quality assurance and patient safety work, is suboptimal. As transparent reporting and analyses of serious adverse events is crucial for learning risk management and preventing adverse events, reporting culture and compliance with guidelines need to be improved substantially for better safety in patient care, risk mitigation and clinical quality assurance.

## Data Availability

The data that support the findings of this study are available from The Norwegian Board of Health Supervision (NBHS), but restrictions apply to the availability of these data, which were used under license for the current study, and so are not publicly available. Anonymized data can, however, be made available with the permission of NBHS by the corresponding author upon reasonable request.
